# A Semantic Sensor Web for Environmental Decision Support Applications

**DOI:** 10.3390/s110908855

**Published:** 2011-09-14

**Authors:** Alasdair J. G. Gray, Jason Sadler, Oles Kit, Kostis Kyzirakos, Manos Karpathiotakis, Jean-Paul Calbimonte, Kevin Page, Raúl García-Castro, Alex Frazer, Ixent Galpin, Alvaro A. A. Fernandes, Norman W. Paton, Oscar Corcho, Manolis Koubarakis, David De Roure, Kirk Martinez, Asunción Gómez-Pérez

**Affiliations:** 1 School of Computer Science, University of Manchester, Oxford Road, Manchester M13 9PL, UK; E-Mails: I.Galpin@cs.man.ac.uk (I.G.); A.Fernandes@cs.man.ac.uk (A.F.); N.Paton@cs.man.ac.uk (N.P.); 2 GeoData Institute, University of Southampton, Southampton SO17 1BJ, UK; E-Mails: jds@geodata.soton.ac.uk (J.S.); oyk@geodata.soton.ac.uk (O.K.); 3 Department of Climate Impacts and Vulnerabilities, Potsdam Institute for Climate Impact Research, P.O. Box 60 12 03, D-14412 Potsdam, Germany; 4 Department of Informatics and Telecommunications, National and Kapodistrian University of Athens, Athens 15784, Greece; E-Mails: kkyzir@di.uoa.gr (K.K.); mk@di.uoa.gr (M.K.); koubarak@di.uoa.gr (M.K.); 5 Ontology Engineering Group, Universidad Politécnica de Madrid, Campus de Montegancedo s/n 28660, Boadilla del Monte, Madrid, Spain; E-Mails: jpcalbimonte@delicias.dia.fi.upm.es (J.-P.C.); rgarcia@fi.upm.es (R.G.-C.); ocorcho@fi.upm.es (O.C.); asun@fi.upm.es (A.G.-P.); 6 School of Electronics and Computer Science, University of Southampton, Southampton SO17 1BJ, UK; E-Mails: krp@ecs.soton.ac.uk (K.P.); ajf3@ecs.soton.ac.uk (A.F.); dder@ecs.soton.ac.uk (D.D.R); km@ecs.soton.ac.uk (K.M.)

**Keywords:** semantic sensor web, application and visualisation, semantic data integration

## Abstract

Sensing devices are increasingly being deployed to monitor the physical world around us. One class of application for which sensor data is pertinent is environmental decision support systems, e.g., flood emergency response. For these applications, the sensor readings need to be put in context by integrating them with other sources of data about the surrounding environment. Traditional systems for predicting and detecting floods rely on methods that need significant human resources. In this paper we describe a semantic sensor web architecture for integrating multiple heterogeneous datasets, including live and historic sensor data, databases, and map layers. The architecture provides mechanisms for discovering datasets, defining integrated views over them, continuously receiving data in real-time, and visualising on screen and interacting with the data. Our approach makes extensive use of web service standards for querying and accessing data, and semantic technologies to discover and integrate datasets. We demonstrate the use of our semantic sensor web architecture in the context of a flood response planning web application that uses data from sensor networks monitoring the sea-state around the coast of England.

## 1. Introduction

Sensor networks are increasingly deployed to monitor the state of the physical environment around us. Over time, a fuller picture of the environment can be built up by analysing the historic values sensed with these devices, and relating these to the dynamically changing current values, thus enabling a better understanding of both current and evolving conditions. For example, consider the benefits of being able to forecast the severity of tidal surges, and the resulting flooding, which have the potential for devastating effects to business and lives. To effectively predict when a surge is going to take place requires gathering data from a wide variety of sources published by independent autonomous providers: sensor networks that monitor the status of the sea provided by research institutions, government agencies, and private companies; weather forecasts provided by national meteorological offices, and companies; and coastal defence information provided by government departments. These are used as inputs to environmental models which predict the future sea-state, and the probabilities that sea defences will be breached or over-topped. Moreover, planning the response to a potential flooding event requires a large number of additional sources to be available (e.g., shipping, traffic, and man-made assets), which can be related to the results of the forecast and the current conditions.

An extensive review of advances in geosensor networks [1] identified the need to integrate sensor network data with existing, large-scale sensors such as remote sensing instruments or large, stationary ocean buoys. Processing the information in real-time using a data streaming paradigm was also stated as a major challenge for geosensor networks. The idea of a *sensor web*, which enables the interoperability of sensor data to support re-use of existing sensor networks, and relating the sensor data with stored data (*i.e.*, historic and contextual data in databases) and graphical sources (e.g., maps, raster, vector), aims to meet these challenges. More broadly, the key features of a sensor web architecture are the ability to:

1. identify relevant sources of data;
2. access sensor data in near real-time together with contextual data;
3. combine and correlate data from disparate sources with differing modalities (*i.e.*, a stream of sensor data with contextual data stored in a database); and
4. enable users and data providers to work with their conceptualisation of the data, *i.e.*, not force users and data providers to use a common data model, particularly as data sources will not be under the control of the users, and could already be publishing their data according to their own conceptualisation.

Similar issues were identified in a recent vision paper [2]. Specifically, the common goals include: support for reusing data originating from autonomous sources; a service infrastructure that supports multiple usage paradigms, viz. request/response, event driven, and resource-oriented; and to enable context sensitive applications that seamlessly integrate environmental monitoring.

In this paper, we argue for the use of semantic web technology to supply rich metadata in a machine understandable format together with a set of services to exploit these semantic annotations to address these issues. We describe a sensor web architecture that enables integration and correlation of multiple heterogeneous datasets, including both live sensor data, historic sensor data, and contextual data (e.g., details of sea defences as provided by the UK Environment Agency’s National Flood and Coastal Defence Database (nfcdd) [3], or the road network as a map overlay). We propose an approach that makes extensive use of semantic technologies (*i.e.*, ontologies [4], rdf [5], sparql [6]), both for data discovery and data integration. This enables users to interact with data within a conceptualisation that they are familiar with. On the other hand, it does not tie the system to one particular model over the data. Indeed, publishers are free to publish their data according to the model in which it is generated. The publisher could already be publishing their data in a particular model. We aim to support the reuse of data in ways that transcend its original purpose.

The services of the semantic sensor web approach build on existing web service recommendations for data access and integration [7], and notifications [8]. Our approach is complementary to the Open Geospatial Consortium Sensor Web Enablement (ogc-swe) framework [9,10] in that existing ogc-swe services (e.g., Sensor Observation Service) can be incorporated into our approach, as well as being used as services on top of our semantic sensor web (as will be described in Section 4).

We demonstrate the use of our semantic sensor web architecture in the context of a flood response planning web application. The application is aimed at a variety of user groups from members of the public to those responsible for identifying and responding to coastal flooding events. The application uses the semantic registry service to discover and dynamically incorporate relevant sources of sensed, modelled, and contextual data according to the user’s role and area of interest. Stored and streaming data services are used to access historic and near real-time sensor data, including sources which may have been semantically combined through data integration services. For example, the application provides an innovative tool for alerting users to the incidence and forecast of flood defence over-topping events.

Section 2 provides details of related work in the areas of sensor web architectures and decision support systems. We motivate the use of ontologies for modelling data in the semantic sensor web in Section 3, and present the network of ontologies used in the flood warning system deployment. In Section 4 we provide a detailed description of the services that form the semantic sensor web and their interactions. Section 5 describes the mechanisms for developing environmental decision support applications that mash-up data from several data sources. Our conclusions are given in Section 6.

## 2. Related Work

In this section we present related work in three areas. First we present related sensor web architectures. We then describe other approaches to providing a semantic sensor web. Finally, we present the state of the art in decision support applications.

### 2.1. Sensor Web Architectures

The term *sensor web* was originally used to describe a wireless sensor network architecture in which the nodes of the network were autonomous and able to react to the data measured by themselves and other nodes in the network [11]. Since then, the term *sensor web* has been used more generally, as it is in this paper, to describe a distributed web service architecture for publishing, discovering, and combining data from multiple sensor networks and related data sources.

One proposal for such a sensor web architecture is the set of data model definitions and web service specifications that comprise the Open Geospatial Consortium Sensor Web Enablement (ogc-swe) framework [9,10]. The three data model standards—Observations and Measurements Schema (o&m) [12], Sensor Model Language (SensorML) [13], and Transducer Markup Language (tml) [14]—provide syntactic data models for representing sensor measurements, the sensors that capture the measurements, and the processing performed on the measurements, respectively. The web service specifications define a service-oriented architecture that provides the functionality to interoperate with sensors and their data across organisation boundaries over the Internet. The Sensor Observation Service (sos) [15] provides the means by which sensor data can be published, allowing other services and applications to request sensor data. The Sensor Planning Service (sps) [16] enables, where it is permitted by the sensor, new tasks to be passed to the sensor. The Sensor Alert Service (sas) [17] and Web Notification Service (wns) [18] provide mechanisms by which services, applications, and users can receive alerts regarding sensor readings.

A reference implementation of the ogc-swe framework was developed in the sany project [19,20]. The sany project provided soap bindings for the abstract service definitions of the ogc-swe specifications. This enables clients to interact with services using standard web service messages (viz. soap) encoded in xml. The sany services also provided a rest interface [21] to the services. This enables clients to interact with the services through a http interface offering the operations get, post, put, and delete. (See [22,23] for a comparison between rest and soap web services.) However, issues of providing a rest-style interface, which focus on resources rather than operations, to a service-oriented architecture were not addressed in the sany project, see Section 4.4 for more details. A central notion of the sany project was the “plug and measure” paradigm whereby sensor networks could simply be deployed in the field and the measurements returned through a standard service interface. However the approach relies on the metadata to describe the sensor deployment having already been stored in the system. The sany project also defined a Cascading Sensor Observation Service for merging data from different sources and performing some post processing of the data, e.g., converting units or computing moving averages [24].

The ogc-swe architecture, and sany implementation, enables interoperability between disparate sensor networks by enforcing a common model and syntax for the data: data is exchanged as xml according to the xml Schema models defined by the ogc-swe standards. The semantic sensor web architecture proposed in this paper supports the independence of the data sources by allowing them to publish their own data models: a particularly important consideration when data is already being generated by sensors owned by autonomous institutions, or being operated by experts from different domains (each of which has its own set of terminology and concepts). The sany Cascading sos supports the merging of data from sos sources. However, it relies on the data conforming to the standard syntactic encoding, o&m gml. The semantic integrator in the architecture described in this paper supports the merging and correlation of data from disparate sources, including those external to our framework (e.g., sos). This approach to reuse transcends the original purpose for collecting the data or our set of services.

The ogc-swe framework does not define a registry service. One option for incorporating such functionality is to use the OpenGIS catalogue service [25]. The OpenGIS catalogue service is based on an attribute-value pair data model over which filters, expressed as attribute-operator-value statements, can be applied. In the semantic sensor web architecture proposed, both the data model (viz. rdf) and the query language (e.g., stsparql) provide a greater level of expressivity. For example, semantic terms drawn from an ontology can be used to refer to spatial geometries besides using coordinates to explicitly specify them.

The European projects osiris and genesis have developed a Sensor Instance Registry (sir) that offers operations for inserting, harvesting and querying information about sensor instances [26]. SensorML is used to describe sensor instances and queries are posed using a combination of spatial constraints, temporal constraints and keywords. Query answering is carried out by using three indices, one for each of the three kinds of criteria allowed. There is another component called Sensor Observable Registry (sor) where semantic information about phenomena observed by sensors can be stored using an ontology. sir can use sor to exploit semantic information to offer better answers to queries, e.g., to determine equivalent phenomena. A discussion of how to link this discovery framework with ogc catalogues and automatically expose resources provided through ogc-swe services to them is provided in [26,27].

ogc-swe also enables the tasking of services using their own mechanism provided through the Sensor Planning Service. We adopt the approach of declarative queries that describe data needs. These are passed down to the sources which can then satisfy the need according to their abilities to answer queries. For example, a query over a sensor network which is able to perform in-network processing can distribute the query into the network to save energy [28].

The Sensor Alert Service and the Web Notification Service provide a custom set of operations to provide limited support for publish/subscribe notifications of events. The semantic sensor web architecture proposed in this paper builds upon the Web Services Base Notification standard (ws-n) [8]. In addition stream query processing languages, such as sneeql [29] and sparql_Stream_ [30], provide support for push-based delivery of sensor measurements according to a declarative description of the data need. Note that the specification of the event service in ogc-swe v2.0 [31] takes a similar approach to the one proposed in this paper. However, our approach uses a single service to publish sensor data for either polling or push-based delivery whereas the ogc-swe 2.0 approach requires two services, the sos and the eventing service.

### 2.2. Semantics in the Sensor Web

The term *Semantic Sensor Web* was coined by Sheth *et al.* [32], who proposed to combine the ogc-swe framework with existing W3C standards for annotating service interfaces and publishing sensor data as rdf. After this pioneering work, many other works have embraced the idea of applying semantic technologies for different tasks related to the discovery and integration of data stemming from autonomous heterogeneous data sources, including the work that we present in this paper. The work presented in [33] uses semantic descriptions of sensor deployments to support discovery and syntactic conversion of the data from sensors for publication into an ogc-swe sensor web. However, the semantic descriptions are not published in the sensor web, which uses the syntactic approaches of the ogc-swe framework for data discovery and publication.

In [34], five challenges are identified for the application of semantics to the Sensor Web. The first of these challenges is the abstraction level in which sensor data can be obtained, processed and managed in general. The second is the need to handle adequately the quality of data. The third challenge is the integration and fusion of data coming from heterogeneous and autonomously deployed sensor networks. The fourth challenge is the identification and location of relevant sensor-based data sources. The final challenge is the rapid development of applications that are based on these types of data sources. Theses five challenges are addressed by the semantic sensor web presented in this work.

There is also some ongoing work addressing the publication of sensor data on the Web, in the form of linked sensor data [35,36], although no standardisation or set of agreed best practices have been achieved so far in this respect.

### 2.3. Decision Support Systems

Computer based *Decision Support Systems* (dsss) are information systems to enable users to make informed choices based on data and forecasts from a wide variety of sources [37]. dsss support a wide range of functionality and capabilities to help planners and managers to assess alternatives and ultimately take appropriate decisions. Increasingly, dsss are offered as web-based or mobile applications. These offer a considerable advantage over traditional dsss in terms of better cooperation capabilities, access to distributed data sources [38], or increased usability [39]. A number of web-based dsss applications have been developed to use data stemming from the ogc-swe framework [31]. However, these are limited by the capabilities of the ogc-swe framework, as described above, and cannot exploit semantic information provided with the data for discovery, integration or linking to related datasets.

The DHI Group have developed Flood Watch [40] and the Mike modelling framework [41] to provide a dss in the context of coastal flooding. These proprietary tools provide real-time forecasts in areas prone to flooding and issue early warnings to flood response managers and the public. They can be used to manage and examine real-time data from a range of external sources, support a range of modelling tools, and include a scenario management tool aimed at carrying out comparative assessments and response planning. Being a closed-source system, however, this framework requires extensive customisation and user input at all levels of the decision making process. The ability to dynamically discover relevant data sources, provided by the semantic sensor web architecture proposed in this paper, and the reliance of a light web-based client architecture instead of a centralised approach is certainly beneficial from the point of view of stakeholder cooperation, e.g., in the emergency response scenario used to motivate our approach. The use of ontologies as a means of mutual understanding between different types of system users in the development and deployment of decision support systems, advocated by this paper, has been successfully tested by the developers of semantically enabled SoKNOS emergency management system [42].

Casola *et al.* [43] describe the architecture of a spatially and sensor type-constrained Early Warning System which combines data from multiple sensor networks and low-level services. The architecture consists of a data modelling and access service, a computing and simulating service, and an alert and notification service. The proposed semantic sensor web architecture aims at ensuring interoperability of different sensor platforms together with contextual datasets and services that operate on the published data, and therefore has the potential to provide the decision makers with more flexible solutions than other highly-customized early warning systems. Jirka *et al.* [44] identify the importance of accessing multi-domain semantically-enabled sensor information for efficient risk monitoring in a number of use scenarios. Our semantic sensor web provides the richer functionality for discovery, data access, and integration identified by Bröring *et al.* [31] as a requirement for disaster management or early warning systems.

## 3. Modelling Semantic Sensor Web Information

A sensor web provides an information space to enable users to share and manipulate sensor and related data. This assumes that users, and publishers of sensor and related data, can express the meaning of the data according to some model of the real world. The ogc-swe approach requires all data publishers and users of the sensor web to use the o&m data model that represents data in xml according to a published xml schema. However, this approach requires that existing data sources, e.g., the channel coastal observatory (cco) sensor network [45] or the national flood and coastal defences database (nfcdd) [3], to map and transform their data to this model rather than publishing their own existing conceptualisation. It does not allow for all the forms of heterogeneity that exist in the sensor web setting, or for the representation of the relationships of the data models of related data sources. It also does not facilitate automated reasoning to classify the data.

A requirement for a sensor web is to support the *ad hoc* responsive evolving use of an information space. The data resources of the information space will contain various forms of heterogeneity, including data modality (*i.e.*, sensed, stored, and graphical), data model (*i.e.*, terminology), and data representation (e.g., relational, rdf, and xml). These need to be reconciled into a coherent conceptualisation for a specific user need. (Note that different conceptualisations are likely to be required for different applications.) We use ontologies to represent the common data model for the information space since they facilitate: (i) describing the different infrastructure services and data sources as well as any domain-dependent information; (ii) having a shared vocabulary to interoperate both across the internal infrastructure services, and between that infrastructure and external sources that adopt alternative approaches, e.g., ogc-swe based ones [9]; and (iii) discovering, accessing, and integrating information that is shared within the infrastructure.

**Figure 1.**
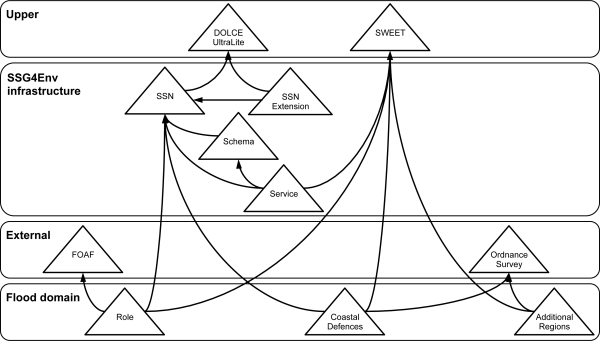
Ontology network for the flood emergency planning scenario. The arrows indicate ontology reuse. Only the bottom layer of ontologies is specific to the flood application domain.

Figure 1 illustrates the ontology network that is used in the flood emergency planning scenario [46]. The ontology network is composed of different ontologies classified in different layers according to whether the ontology can be used to represent: domain-specific information required for the scenario, information required for the infrastructure, or upper-level information used to facilitate interoperability among the other ontologies. These ontologies satisfy the following knowledge representation requirements extracted during the development of the architecture and of the scenario prototype.

- To represent sensor networks and their observed information about properties of certain features of interest. This is covered by the *SSN* ontology [47], developed by the W3C Semantic Sensor Network Incubator Group [48]. The *SSN* reuses the *DOLCE+DnS UltraLite* upper ontology [49]. The *SSN* is broadly based on the ogc-swe o&m model.
- To represent observation collections (included in the ogc-swe o&m specification but not in the *SSN* ontology), summary data for observation collections, measurement property values, and units of measurement. This is covered by the *SSNExtension* module, an extension of the *SSN* ontology that covers these requirements.
- To represent schema metadata about relations and relational streams. This is covered by the *Schema* module that extends, and corrects, an ontology for relational data and schema components [50].
- To represent the web services provided by the infrastructure and the datasets they provide access to. This is covered by the *Service* module that reuses the *SWEET* upper ontologies [51] and includes concepts from the ISO19119 standard on geographic information services [52]. Note that the *Service* ontology could be related to semantic web service ontologies such as owl-s [53] or wsmo [54] to support the automated orchestration of services.
- To represent the geographic and administrative regions of the south coast of England. This is covered by the *Ordnance Survey* ontologies [55], which include the regions from Great Britain, and by the *Additional Regions* ontology, which includes other regions needed in our scenario.
- To represent those features of interest and their properties that are specific to the flood emergency planning scenario. This is covered by the *Coastal Defences* ontology.
- To represent the different roles involved in a flood emergency planning scenario. This is covered by the *Roles* ontology.

All the ontologies have been implemented in the owl ontology language [4], using an ontology engineering tool, and are published on the Web [46]. These ontologies may be updated to meet new requirements in the future; either by upgrading the model represented in the ontology (in the case of ontologies developed by us), or by replacing an ontology with a newer version (in the case of external ontologies). In both cases, new requirements will lead to a new ontology development cycle that will end with new versions of the affected ontologies.

Regarding the adaptability of the ontologies presented here, while some of them are specific to the flood warning scenario, e.g., *Role*, the architecture proposed in the next section is generic. Thus, it can be adapted to other situations by replacing the flood domain ontologies. Note that if an ontology exists for the new application domain, then it is a straightforward process to replace the flood domain ontologies with the ontology for the new domain. Otherwise, a new ontology would need to be developed. This requires a domain expert, who knows the terminology and relationships of the concepts in the domain, to work with an ontology engineer.

## 4. Sensor Web Architecture

**Figure 2.**
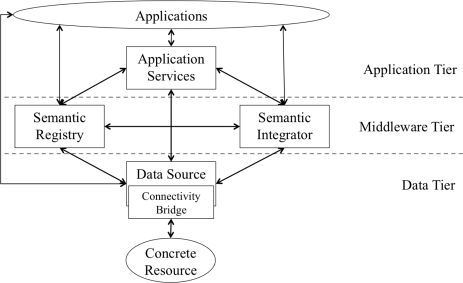
The service types, and their interactions, that form a semantic sensor web. Note that the Data Source service type collectively represents the three types of data service (see Section 4.2).

This section defines a semantically enabled sensor web architecture (depicted in Figure 2). The architecture supports:

1. discovery of data sources and services based on their content and spatiotemporal coverage;
2. accessing and manipulating sensor and related data in near real-time;
3. on-the-fly integration of multiple heterogeneous sensor and stored data sources; and
4. multiple conceptualisations of data.

The core service types of the architecture (viz. Data Source, Semantic Registry, and Semantic Integrator) form a service-oriented architecture which supports orchestrations that combine the functionality of services, *i.e.*, service instances are used as building blocks to construct more feature-rich interactions of the services. These services expose their capabilities in terms of a collection of reusable interfaces (specified in Table 1), which have been defined using wsdl [56] and interchange soap messages [57].

The Application Tier of the architecture provides more domain-specific (e.g., running forecast models over the data as will be described in Section 5.1) or user-oriented functionality (e.g., transforming the presentation of data into a form that is easily processed by user applications). The latter case aims at supporting the increasing trend in decision support systems for rapid development of web-based applications, including mash-ups. Typically, web application developers expect services to offer a rest interface [21], and potentially a sparql endpoint [6]. The service definitions do not preclude implementations from exposing such an interface. However, the High-Level Application Programming Interface (hlapi) service is defined so that it can be configured to exploit the ability to orchestrate the core services whilst exposing a rest interface.

**Figure 3.**
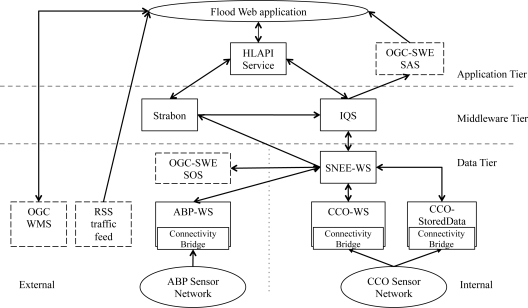
Deployment of semantic sensor web service instances to satisfy the flood surge scenario. Registration interactions have been omitted for clarity of presentation. All services register with the Strabon service, a registry service instance.

To ease the presentation of the architecture, we draw examples from an environmental decision support scenario for a coastal flood surge. The semantic sensor web service instances deployed for this scenario are given in Table 2. The interactions of these service instances, together with sample ogc-swe services, are depicted in Figure 3. The scenario consists of data being published by a variety of sources: some using the services defined as part of the semantic sensor web architecture. For example, the cco-ws service publishes the sensor readings generated by the cco sensor network [45]; the abp-ws service publishes sensor readings about tides, waves, and weather conditions generated by a sensor network deployed by the Associated British Ports authority [58]; and the cco-stored service publishes stored information relating to the cco sensors such as their associate storm threshold value. Others are ogc defined services (e.g., Web Feature Service, sos, and sas), while others are *ad hoc* web feeds (e.g., traffic report rss feed). Note that the instance of the architecture deployed for the flooding scenario makes extensive use of the ontology network presented in Figure 1. However, the architecture does not consider the problem of ontology maintenance.

The following sections describe the principal capabilities supported by the semantic sensor web architecture and provide details of the services which provide the functionality. Full details of the interfaces and services defined by the architecture are available in [59].

### 4.1. Registering and Discovering Datasets

The semantic registry service supports the registration of services and their datasets through the *Registration* interface. The deployed registry service, Strabon (Table 2), accepts rdf documents describing the interfaces supported by the service, and the spatiotemporal and thematic description of the datasets. These semantic descriptions are expressed with respect to the network of ontologies [46] depicted in Figure 1. For example, descriptions of sensor networks and corresponding observations refer to the Semantic Sensor Network (ssn) ontology [47]. Note that the registry service implementation is not limited to using terms from the ontology network.

The semantic registry supports the discovery of datasets by answering queries posed through the *Query* interface. The service implementation accepts queries expressed in stsparql: a sparql extension which has additional support for spatiotemporal constraints [60]. This enables dataset and service discovery based on the spatiotemporal and thematic coverage of the data, expressed in terms of concepts from the ontology network, rather than simply in terms of the functionality offered by the service. For example, an emergency response planner who is responsible for a specific region, say the Solent—the strait of water separating the Isle of Wight from the south coast of England—can specify their region using the relevant concept from the ontology network, viz. AdditionalRegions:Solent. The ontology concept contains a definition of the Solent region as a polygon represented using the Well-Known Text (wkt) format [61]. Alternatively, an environmental scientist who focuses on an area of coastline that exhibits particular features and which spans different regions can specify the exact region directly.

**Figure 4.**
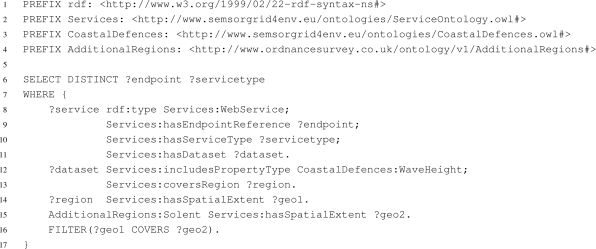
Declarative query to the Strabon registry service in the stsparql language. The query discovers service endpoints and their type for data sources providing wave height measurements for the Solent region.

The queries posed to the registry service can request any information that is stored in the registry. The information returned is either in the form of sparql bound variables or an rdf document, depending on the query posed. Thus, the client can retrieve only the endpoints of selected services or all the information stored in the registry about a service. Figure 4 shows an example query posed to the Strabon registry service to discover the endpoint reference and service type of all data sources for the Solent that provide data about wave height sensor readings. Lines 13 and 14 declare the area covered by the dataset, while line 15 declares the region of interest to be the Solent. The FILTER on line 16 ensures that the dataset covers the Solent region. The results are returned as sparql bound variables.

#### Discussion

The functionality offered by the semantic registry service goes beyond that offered by ogc-swe. An ogc-swe service may use the OpenGIS catalogue services specification [25] for discovery purposes. The common query language developed for interoperability in this specification is based on an attribute-value pairs data model and attribute-operator-value atomic expressions in a Boolean query. In our approach we are more permissive, in the sense that the architecture allows any query language to be used. The architecture is agnostic to the actual language used by the registry instance, which allows for implementations that use stsparql [60], geosparql [62], or sparql-st [63]. In the implemented Strabon registry service [64] the user can use stsparql, which is a declarative query language. The queries are evaluated over the stored metadata using thematic and spatial criteria instead of being restricted to attribute-operator-value methods that ogc-swe provides. This is more general in principle. Also, the concrete realisation of the semantic registry service by any rdf store offers a semantics-based data model which is more expressive than an attribute-value pair data model. Additionally, in our approach the user can use real-world concepts, e.g., semantic terms that are defined in a dataset exposed as linked data such as LinkedGeoData [65], to refer to spatial geometries besides using coordinates to explicitly specify them.

geosparql is being developed in an ongoing ogc standard working group [66] for representing and querying geospatial data expressed in rdf. Earlier contributions in this area include [63,67,68] and our original paper on stsparql [60]. stsparql [60,64] has many commonalities with the recent ogc work on geosparql. Both approaches represent geometric objects as spatial literals and may be encoded in various formats like gml, kml, or wkt. In both approaches, basic spatial functions, spatial predicates, and functions that support spatial analysis, are mapped to extension functions that may be used in the SELECT clause or the FILTER conditions of a query. In geosparql, basic spatial functions and spatial predicates are also mapped to rdf properties. This allows geosparql to be easily combined with other representational frameworks that formalise qualitative spatial reasoning (e.g., rules as in [69]). A point where the two languages differ is that geosparql offers a minimal ontology that can be used for representing features and geometric objects. In our approach, we impose very minimal requirements to semantic web developers; all they have to do is utilise a new literal datatype and they are free to define and use their own spatial ontologies. The approach followed by [70] is also the same, although the authors concentrate more on indexing and query processing and less on defining a complete geospatial extension of sparql.

### 4.2. Publishing Datasets

The data source component depicted in Figure 2 denotes three distinct types of data service: (1) streaming data services that enable access to sensor data as continuously updated streams of values; (2) stored data services that enable access to data stored in repositories, such as a relational database or a triple store, including sensor deployments that load their readings directly into a database; and (3) external data services that enable access to data via some defined service interface, e.g., an ogc Web Map Service for publishing map images (ogc-wms [71]), or an ogc-swe sos [15].

#### 4.2.1. Streaming Data Service

A streaming data service is defined as an extension to the Open Grid Forum (ogf) web service recommendation for data access and integration (ws-dai) [72]. Such a service provides the *Service Metadata* interface to enable clients to discover metadata about the service and the datasets that it makes available. The metadata is returned as an xml document that conforms to the ws-dai standard and has been extended to contain an rdf description of the service and its datasets, using terms from the network of ontologies depicted in Figure 1. Registering a streaming data service simply involves sending the metadata document, containing the rdf description, to the *Registration* interface of the registry service.

In its most basic form, the streaming data service makes the readings from a sensor network available as a stream of data values through one, or both, of the *Data Access* or *Subscription* interfaces. These enable clients to access the readings in the time series as they become available. In the case of the *Data Access* interface, the client must poll for the value. The rate at which the client polls for new values can be configured based on the information contained in the metadata, e.g., the expected rate of the data stream. In the case of the *Subscription* interface, the client must expose the *Notification* interface for receiving notification messages that new sensor readings have been generated and published. The publish/subscribe mechanism uses the oasis Web Services Base Notification (ws-n) standard [8].

**Figure 5.**
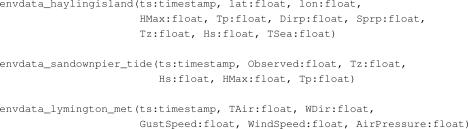
Example schema of the streams generated by the Channel Coastal Observatory wireless sensor network deployment; published as a streaming data service (cco-ws).

As a specific example, consider the sensor network deployed by the Channel Coastal Observatory (cco) [45] around the coast of England and published through the cco-ws streaming data service (Table 2). This is an active wireless sensor network consisting of 43 sensor nodes deployed at strategic locations, many along the south coast of England. Each node in the network generates its own stream of data values named after the location of the sensor node. There are three distinct types of stream measuring properties of waves, tides, or meteorological conditions; an example of the schema of each type of stream is shown in Figure 5. There are 24 nodes measuring wave properties with the same schema as envdata_haylingisland, 7 nodes measuring the properties of tides with the same schema as envdata_sandownpier_tide, and 12 nodes measuring meteorological values with the same schema as envdata_lymington_met. Since the sensor nodes in the cco sensor network have a fixed sensing period, viz. they take a new reading every 10 minutes, the rate of the streams is published in the metadata document. This enables clients to configure the rate at which they poll for data.

Streaming data services can also provide a *Query* interface. The *Query* interface supports clients in providing a declarative description of their data needs as a query. The *Query* interface does not specify the language in which the query is expressed; this is available in the service metadata. This allows services to support a query language suitable for their data, e.g., sneeql for continuous queries over streaming relational data [29]. The response to a query operation is the generation of a data stream whose values satisfy the query. This new stream is made available either through the *Data Access* or *Subscription* interface. Finally, the service can also offer the *Integration* interface to support queries involving more than one data source, e.g., another streaming data service. In the case that an external data source is used, the *Notification* interface can be provided to support subscriptions to external sources. An example of this more feature-rich streaming data service is made available by the snee-ws service (Table 2), which uses the snee query engine to process streaming data queries [28]. The deployed snee-ws service is configured to use the streams published by the cco streaming data service as its data source. Thus, it provides a query interface to that data. This enables the integration service (Section 4.3) to pose queries over the streams published by the cco-ws, e.g., the 24 wave streams can be merged into a single wave stream using a UNION query. snee-ws is also capable of evaluating queries over a mix of streaming and stored data services with a well-defined evaluation semantics (details of a motivating case for such queries are given in Section 4.3).

#### 4.2.2. Stored Data Service

The stored data service enables access to data stored in repositories such as relational databases, xml stores, or triple stores. This can include retrieval of values from a sensor network source which are first stored in a database, access to the historic values from the data stream, or data associated with the deployment of the sensor network, e.g., the location of the sensors or associated threshold values. We have adopted the ogf ws-dai [7,72] recommendation for the stored data service and the reference implementation based on ogsa-dai [73].

Clients can discover the metadata associated with the stored data service through the *Service Metadata* interface, which returns an xml document describing the service and the datasets. The stored data service enables declarative queries to be evaluated over its data content through the *Query* interface. Since the answer to a query can be potentially large, the answer can be broken into chunks that are retrieved through the *Data Access* interface.

The metadata associated with the cco sensor network and the history of sensor readings is published as a database through the cco-stored stored data service (Table 2). One of the relations published in this database associates a storm threshold value with each of the sensor data streams and has the schema

locations(id:int, latitude:decimal, longitude:decimal, storm_threshold:decimal, deployed:timestamp).

Section 4.3 will show how the stored storm threshold data can be combined with the live sensor data to detect an over-topping event.

#### 4.2.3. External Data Services

The specification of the streaming and stored data services are targeted at specific types of data, *i.e.*, time series generated by a sensor network and data stored in a database, respectively. For other types of data, e.g., map features and bitmap images, there are existing standards such as the ogc Web Feature Service (ogc-wfs) [74] and the ogc Web Map Service (ogc-wms) [71] that are suitable. These can be used in our conception of a semantic sensor web by manually registering a semantic description of the service and its data with the registry service. A web page interface to the Strabon registry service has been made available for this purpose (http://www.semsorgrid4env.eu/services/registry-service/Store). Indeed, the use of ogc services is the approach adopted with the output of the environmental models for forecasting sea-state, which make available a map image of the raster grid as an ogc-wms (see Section 5.1). Our approach to external data services also supports the use of other sources of time series data such as made available by an ogc-swe Sensor Observation Service [9]. Note that these must also be manually registered in the registry.

#### 4.2.4. Discussion

The functionality offered by the streaming data service combines the data publishing functionality offered by the ogc-swe sos and sas services, whilst offering richer data processing and filtering capabilities. The sos only supports clients polling for data while the sas supports the pushing of data to clients that have subscribed. Both forms of interaction are supported by the streaming data service. By exposing the *Data Access* interface, the streaming data service supports clients in polling for sensor readings. By exposing the *Subscription* interface, the streaming data service is able to push sensor readings to subscribers in a timely fashion. The streaming and stored data services do not prescribe a data model or format for the data published, unlike the ogc-swe services which requires that data conforms to the o&m syntactic model. This provides a lower entry requirement for data publishers as they only need to describe their data, not map or transform it to some common data model that may not accurately capture their data. Finally, the streaming and stored data services offer the possibility of supporting declarative queries by exposing the *Query* interface. A declarative query language, such as sneeql [29], provides the possibility to correlate stream values, both within the stream and across streaming and stored sources, and compute aggregates such as an average. This goes beyond the simple attribute-operator-value filtering offered by the sos.

### 4.3. Data Fusion: Integrating Heterogeneous Datasets

A key requirement for a sensor web is the ability to combine data from multiple sources. Existing approaches rely on either the end user manually combining datasets in a spreadsheet, or using data fusion or mash-up approaches which simply juxtapose datasets from multiple sources. The latter cases rely on the end user to visually interpret the result and the resulting data set has no coherent semantics. Such an approach can result in useful data but can be more laborious.

A key ability of the semantic sensor web is integrating and correlating data from heterogeneous sources: both in terms of the modality of the data (*i.e.*, streaming and stored), and the representation of the data (*i.e.*, the schema used). Through the *Integration* interface, the integration and query service (iqs Table 2) supports the creation of a *virtual* data source in which the data from multiple physical data sources appear to co-exist in a single data model. The virtual data source can be queried through the *Query* interface and query answers retrieved through either the *Data Access* or *Subscription* interfaces.

The iqs is configured to expose an ontological view over a set of data sources through a mapping document. The mapping document relates the data conceptualisations exposed by the data sources to a global data model. The mappings are expressed in terms of selections and transformations over the data sources, and can be created either manually or with the help of a mapping tool. For example, the iqs service deployed for the flooding application exposes the data published by the cco-ws service and the abp-ws service using the *SSN* ontology. Users and applications can pose queries expressed in sparql_Stream_ over the ontology. These are transformed, according the relationships expressed in the mapping document, by the iqs into queries over the data sources. The generated queries are then passed to the snee-ws service with the answer streams flowing back. Full details of the mappings and the semantic integrator implementation can be found in [30].

**Figure 6.**
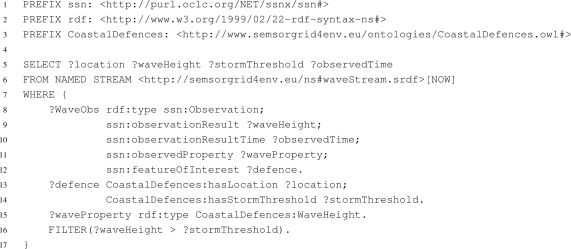
Declarative query in the sparql_Stream_ language which characterises an over-topping event over an ontological observation model for sea-state readings. The event is characterised by the measured wave height being greater than the associated storm threshold value for a specific sensor.

A motivating use case for the integrator, drawn from the flood surge application, is to expose sensor readings and associated metadata for the south coast of England. A user, most likely a domain expert, creates the virtual source by providing a mapping document to the iqs through the *Integration* interface which states how to relate the source streams of the cco-ws and the abp-ws together with the tables of the cco-stored to the ontological model of the *SSN*. The integrated data source is automatically registered with the registry service, making it available to all users. Any user can then pose queries over the resulting data source to explore aspects of the data, or indeed be informed of interesting events as characterised by queries over the integrated source. For example, it is desirable to be informed when a sea defence has been over-topped by a wave. This can be determined by a query that relates the wave height readings from the sensor network with the associated threshold value stored in the database. The query over the integrated observation model is shown in Figure 6 expressed in sparql_Stream_. Line 16 of the query relates the data stored in the cco-stored database with the current sensor reading from the cco-ws, the keyword ‘NOW’ on line 6 limits the sensor readings to the current time-point. The query is evaluated by posing a translated query to the snee-ws distributed query processing service. The snee-ws service poses a query to the locations table of the cco-stored data service and correlates the results with the streams consumed from the cco-ws and abp-ws.

Note that the new data stream generated by the query in Figure 6 can be used to inform users whenever such an over-topping event has taken place. For example, it can be used as an input feed to an alert service such as the ogc-swe sensor alert service [9], as depicted in Figure 3.

#### Discussion

The ogc-swe architecture does not have a service specifically aimed at integrating data from multiple data sources. In the sany project, a Cascading Sensor Observation Service [24] was defined and implemented to support the following use cases: (i) to merge streams and republish the result; (ii) to wrap legacy data sources so that they can be accessed through the sos interface; (iii) to compute simple transformations on the data, e.g., unit conversion or calculating moving averages; (iv) to perform load balancing across deployed services; and (v) to replicate data. The first three use cases are covered by the semantic integration service, except that the semantic integration service can also perform transformations between different views of the data, *i.e.*, from the many views of the data publishers to the integrated ontological view exposed by the integration service. Use cases (iv) and (v) are deployment issues rather than architectural decisions. A deployment of the semantic integration service may choose to replicate its service deployment across several machines and it may choose to replicate the source data or fetch it on-the-fly.

### 4.4. HLAPI: Observation Linked Data Service

The previous subsections have presented the core web services that form a service-oriented architecture, specifically those which comprise the data and middleware tiers of the semantic sensor web architecture (Figure 2). The focus of those services is to provide programmatic access to the data for the creation of orchestrations to enable discovery, publication, and integration of data. The top tier of the semantic sensor web architecture provides services which are domain-specific and user-facing. An example of such a service is the High-Level Application Programming Interface (hlapi) service (Table 2). The focus of the hlapi service is to support higher-level tools, particularly web-based applications and mashups. For example, to enable time series sensor data to be presented in a mash-up together with other contextual datasets. This is achieved through a linked data [75] approach to publishing sensor observations, received from the data services and semantic integrator, in combination with rest conformant representations specific to the geographic and environmental domain.

While it is possible to send and receive soap messages from code running in a web browser using a library such as CXF [76], this approach is, in general, not followed in mash-up development. Web application developers typically expect to retrieve data through a rest interface [21] supporting the four http operations, viz. get, post, put, and delete, that are supported in the execution environment provided by a web browser; services following this pattern are deemed “restful”. To make full use of the semantic content provided through the semantic sensor web while exposing data in a manner consistent with rest design, it is also desirable for an interface to follow the principles of linked data [75]. That is: (1) use uris as identifiers for resources; (2) use http uris so that resources can be looked up; (3) provide meaningful standards-based representations of the resources when they are looked up containing links to more data—also known as the follow-your-nose principle; and (4) include uri links to related resources to enable discovery through link navigation.

In the environmental area, many mash-ups and web applications are further characterised by an approach by which datasets are overlaid on a base map with each dataset displayed being transformed into a layer (as depicted in Figure 10 and explained in Section 5.2). To create these map layers, the web application needs to access the data through an interface that is supported in the restricted execution environment provided by a web browser, and in a format that can be converted to a layer in that restricted execution environment (e.g., a geojson array, or wfs gml). These formats are, when accessed through a rest interface, additional representations that complement those provided as linked data (viz. rdf).

Applying these complementary rest and linked data approaches over the ontology network described in Section 3, a High-Level api (hlapi) for sensor observation data has been developed [35,77,78]. It enables web applications to access and navigate the data (practising the follow-your-nose principle) using resources comprising the observations, sensors, and time-series collections of observations grouped by the property being observed, the gathering sensor, and temporal boundaries. Links are made and maintained between collections and observations—a single observation is likely to be included in several collections, and a collection may in turn be a constituent of several higher-level collections—and to externally defined linked data sources, e.g., relating sensor positions to geopolitical boundaries, or observed properties to their definitions in the domain. Representations include those needed for linked data applications (viz. rdf, also via sparql), for use within geographic applications and for layer generation (viz. o&m gml, geojson, wfs gml) and for general human-readable browsing (viz. html). The hlapi is implemented as a service which generates the structure of resources and links between related data resources according to the rules provided in the service configuration. This has two main parts: the alignment of the incoming data streams with a single data model, which is directly inherited from the integrator service (Section 4.3) when that is the providing data service; and the declaration of an api such that resources are appropriate to the specific data being published, e.g., defining collections of manageable size derived from observation frequency.

**Figure 7.**
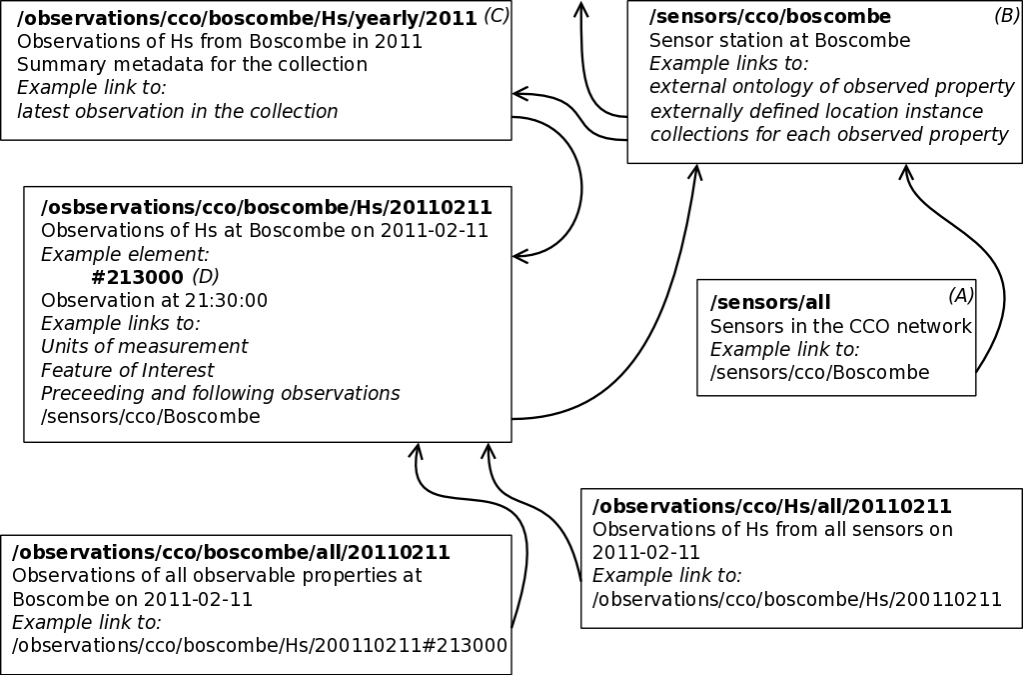
A selection of resources and links provided by the cco deployment of the hlapi (not comprehensive).

For example, to support mash-up developers in the reuse of sensor data published by the cco sensor network (Section 4.2), we have instantiated a hlapi service to provide access to the observations published by the virtual data source available from the integration service. The deployed hlapi service automatically generates uris for sensors and each observation as data is received from the integration service. It also dynamically organises, and creates as required, the collections to group observations according to their location, the property that is measured, and the time at which it was measured. A selection of resources from this example deployment are shown in Figure 7. A semantic web client might initially access the hlapi service using the uri for all sensor resources (*A*) (also Table 2). The client can follow the link to the Boscombe sensor resource (*B*) and request an rdf representation of the Boscombe sensor resource. This representation would contain a semantic description of the sensor in terms defined by the ontology network: including its capabilities, location, and annotated links to collections to which it has contributed observations, such as an annual collection of wave height measurement (*C*). The latest observation, say 9.30 pm on the 11*th* February 2011 (*D*) is accessible through a link. Alternatively a client may receive a link to a specific observation, such as (*D*). From this resource, the client can follow links to preceding or following observations of wave height from the Boscombe sensor, or to collections of which the observation is a constituent. The rdf representation is best used for semantically navigating the dataset due to library and tooling support. However, once the desired resource is located, the client can request any representation, e.g., geojson to create a layer, through content negotiation.

#### Discussion

Beyond an early prototype of the API design outlined in [35], there have been multiple proposals for exposing sensor related information as linked data, each with differing motivations and foci: automated conversion from ogc standards and services [36], alignment with foundation ontologies [79], publishing linked sensor locations and attributes [80], sensor discovery over linked data [81], and integration from multiple sensor sources into a single service [82].

While the hlapi touches upon the issues raised in several of these works, the primary focus during its development was instead the design and deployment of APIs that are accessible and relevant to a developer working in the domain, on linking the semantics of the domain to observations so they can be reused in web applications and mashups, and on utilising the semantics of the domain model to simplify the configuration and deployment of services.

The sany architecture [20] outlines an abstract resource model which can be used to align ogc-swe services with rest practises, most recently leading to a proposal for a restful interface to sos services [83]. The hlapi, while taking a similar approach to restful publication of resources and representations, internally uses an rdf model as its primary representation rather than the provision of resources through an extension of ogc-swe services. This enables the hlapi to preserve and publish resources based upon the semantic structures provided by the semantic sensor web architecture, and to exercise semantic mappings, using the same domain ontologies, to simplify configuration of hlapi deployment.

## 5. Interacting with the Semantic Sensor Web

This section presents the interactions of applications with the semantic sensor web architecture presented in Section 4. We first consider the generation of flood warnings based on the readings from the sensor networks and other sources of data. The approach uses environmental modelling techniques to predict the changes in sea-state for a period of time in the future based on the most recent sensor readings. The results are themselves published as (external) data sources in the deployed semantic sensor web. We then describe a web application that has been created using the services and functionality provided by the semantic sensor web.

### 5.1. Forecasting Flooding Events

Environmental models that forecast the behaviour of the sea-state have the potential to predict future flooding events (within given error margins): specifically where, when, and how severe they may be. These models are computationally expensive to run and depend on data from a wide variety of data sources including sensor data, weather feeds, and data stored in databases. In the context of forecasting flooding events and planning a response, three environmental model services have been developed. These models forecast the sea-state, likelihood of over-topping or breaching flood defences, and the water levels on the flood plains. The output of the models are exposed to applications using the semantic sensor web as layers published by ogc web mapping services (ogc-wms) [71] which have their semantic descriptions registered with the registry service.

The first service models the mean wave height and sea level for the next eight hours. The model uses sea-state data from the cco sensor network together with meteorological data as input. The result is a finite element mesh which predicts the sea-state (level and wave heights) at each point on the mesh and is represented as a raster grid. The raster grid output format (GeoTIFF) is exposed as a collection of layers distinguishable by their order of precedence and a timestamp through an ogc-wms.

The second modelling service uses the output of the sea-state forecast model together with information stored about sea defences in databases such as the National Flood and Coastal Defence Database (nfcdd) of the UK Environment Agency [3]. The input data is processed using an empirical over-topping formula [84] to predict the probabilities of sea defences being over-topped. The output of the model is a collection of ESRI ASCII Grid files which can be served by an ogc-wms.

The final modelling service predicts the depth of water on the flood plains in the event of an over-topping event. It runs the LISFLOOD-FP inundation model [85] using the tidal water levels provided by the sea-state modelling service. The output is a raster grid showing the maximum forecast flood level for each cell at each time point. The result is again made available as map layers through an ogc-wms.

### 5.2. Emergency Response Web Application

**Figure 8.**
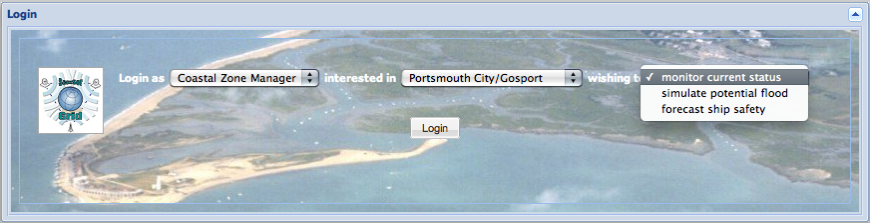
Screenshot of the login screen for the web application.

**Figure 9.**
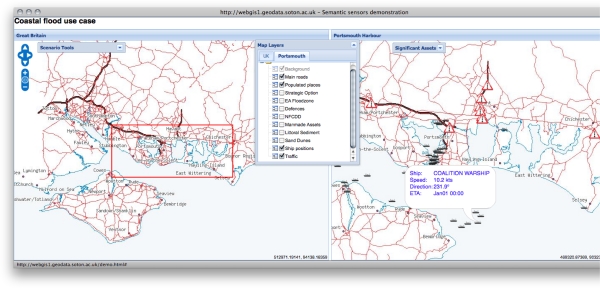
Screenshot of the initial layers displayed for the role *Port Authority* the region *Portsmouth City/Gosport*, and the task of *Forecast ship safety*.

**Figure 10.**
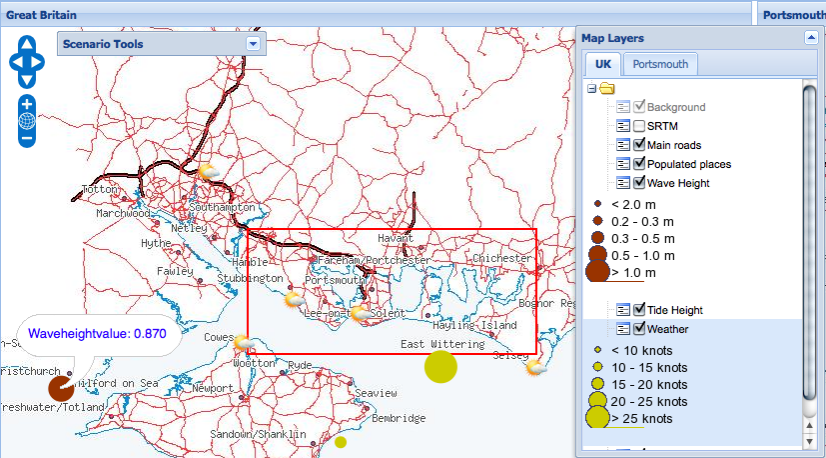
Screenshot showing layers generated from sensor readings.

**Figure 11.**
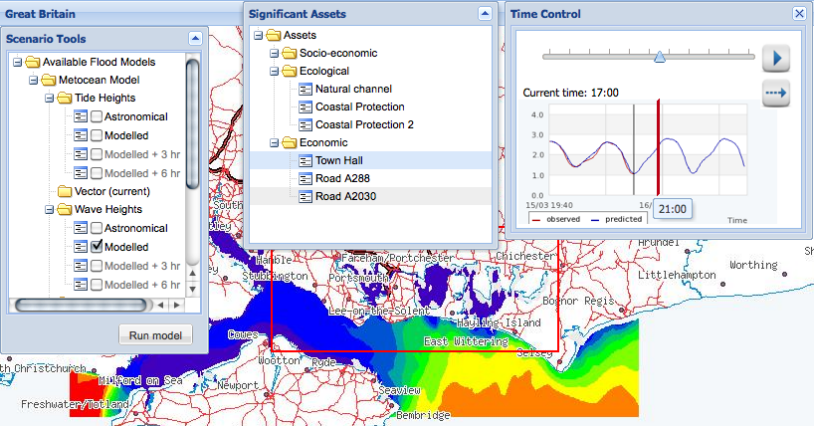
Screenshot displaying the output of the wave height flood model (Section 5.1).

Tidal surges and the resulting flooding have the potential for devastating effects to businesses and lives. A wide range of users, including emergency response planners, harbour masters, and members of the public, need to be supported in interacting with the data in these situations. We have developed a web based application, *i.e.*, one that executes in the limited environment offered by a web browser and only permitted to make http calls, which enables users to discover data sources based on their spatiotemporal and thematic content, and to juxtapose that content as layers on a map. Screenshots from the application are shown in Figures 8, 9, 10, and 11, and will be discussed in the remainder of this section.

The application exploits the functionality provided by the services of the presented semantic sensor web: specifically the ability to discover datasets and services, and to orchestrate the services to integrate heterogeneous data sources and dynamically publish the resulting dataset through a rest interface. For example, the application can interact with data in native web formats, such as geojson, by using an appropriately configured hlapi service instance (Section 4.4) to return observations through a rest interface. In turn, the hlapi service instance can use the semantic integration service (Section 4.3) to retrieve data stemming from multiple heterogeneous data sources, both in terms of modality and data model, as a unified virtual data source which presents its data as instances of an ontology.

In this section we describe the web application and the issues faced in its development. The application is an exemplar of the type of mash-up that can be developed on top of the semantic sensor web. The web application is available from http://www.semsorgrid4env.eu/services/dynamic-demo and http://www.semsorgrid4env.eu/services/static-demo. The latter provides a more stable demonstration interface highlighting stable functionality, while the former aims to be as dynamic as possible, at the potential expense of stability/usability.

#### 5.2.1. The Solent and its Users

The Solent region is the area surrounding the Isle of Wight off the south coast of England. This region has a complex tidal and wave pattern which generates a demand for sea-state forecasts. It is a busy shipping area servicing the ports of Southampton and Portsmouth with passenger ferries, cargo ships, and military vessels as well as a large number of recreational water users. The coastline comprises a variety of built up areas such as the cities of Southampton and Portsmouth, and is home to a number of sites with statutory protection, including special areas of conservation. As a consequence, there is a wide variety in the types of users who are interested in interacting with the data about the region made available by the semantic sensor web. Table 3 illustrates the types of user groups and their interests.

#### 5.2.2. Application Characteristics

The estuarine surge and coastal flooding emergency response web application is built on top of the semantic sensor web architecture using the open source mash-up development tool ExtJS [86] and its geospatial interface extension [87]. The web application is based around the standard gis approach of displaying data contextually as layers on a map. In order to generate these layers we make use of the OpenLayers toolkit [88]. OpenLayers provides a JavaScript api that can be executed within the web browser execution environment, and by which data can be converted into layers for displaying on a map.

The web application aims to support a variety of users, from professional emergency response planners to members of the public, in gaining vital information about a flooding event. This requires dynamically retrieving a variety of data sources and supporting user interaction with these. The typical interaction model is first to discover relevant sources of data based on the spatiotemporal and thematic coverage by querying the registry service (Section 4.1). The user can then select which sources should be added to their display. This can involve using data directly from the original data sources (e.g., the data resulting from the flood models in Section 5.1), using a processed version of the data which is appropriately presented for inclusion in a web application (e.g., data represented as a geojson array from the hlapi service described in Section 4.4), or an integrated source of data which combines the data from a variety of data sources (*i.e.*, data from the integration service described in Section 4.3).

In the rest of this section, we describe the interactions of the application with the semantic sensor web architecture by following a use case for the port authority user (Table 3).

#### 5.2.3. Application Description

Users access the web application through a login screen, shown in Figure 8, which allows them to select their role, the region they are responsible for, and the task that they wish to conduct. These are presented as options in drop-down selection boxes which are populated with terms from the ontology network [46]. For example, the choice of role comes from the concepts in the *Role* ontology. This provides an initial characterisation of the data that is relevant for the interaction, *i.e.*, the values provided parameterise the queries sent to the registry in order to discover relevant data sources. For the example of the Queen’s Harbour Master at Portsmouth, they would select their role as *Port Authority*, their region as *Portsmouth City/Gosport*, and their tasks as *Forecast ship safety*. These options parameterise the query sent to the registry service to discover relevant data sources based on the spatiotemporal and thematic coverage.

The result of the login process is a screen presenting the user with two map views side-by-side (shown in Figure 9), based on the region selected, viz. *Portsmouth City/Gosport*. The left pane displays a zoomed-out map providing context while the right pane displays a zoomed-in map on the region selected. Both maps are superimposed with layers presenting data from a variety of sources that satisfy the queries sent to the registry. The available layers are shown in the *Map Layers* pop-up window, from which the user can select the layers that they wish to be displayed on each map. Initially the context map in the left pane shows the populated areas and the main roads. The detailed map in the right pane includes the same background layers and also displays two additional layers providing details of shipping in the region and traffic alerts respectively. The shipping information is displayed, with a ship icon indicating the position of each vessel, from a streaming data source publishing data obtained from the automatic identification system (ais) network used to track ship positions [89]. The user can discover details of the vessel name, heading, and speed in a pop-up balloon by hovering their cursor over the icon. Similarly, the traffic alerts, shown as warning triangles, are displayed from an external rss web feed.

The *Map Layers* pop-up window shows all possible layers that can be chosen for display, *i.e.*, those that were discovered by the queries to the registry service. The majority of the layers present information located at a specific point in space, *i.e.*, sensor readings. The screenshot in Figure 10 shows a pop-up balloon displaying the latest wave height reading from a node in the cco sensor network. This value is provided by an instance of the hlapi service (Section 4.4) which in turn obtains it from the sensor data stream produced by a query to the virtual data resource published by the integration service (Section 4.3). The interpretation of the size and colour of the circles displaying sensor readings are provided in the *Map Layers* window. Other layers that can be selected display weather readings coming from the deployed sensor network (published through the cco-ws and displayed as yellow circles), forecasts from public meteorological web services (displayed as weather symbols), and details of flood defences coming from stored data sources such as the UK Environment Agency’s National Flood and Coastal Defences database (nfcdd [3], not shown).

The screenshot in Figure 11 shows a model layer presenting the forecast wave heights as generated by the service described in Section 5.1. The result of the model is superimposed on the sea with different colours (shading) depicting regions with different wave heights. The choice of model to display is controlled by the *Scenario Tools* pop-up window and the time point in the model is controlled by the *Time Control* pop-up window. Users can select an appropriate model and forecast period (Astronomical, Modelled nowcast, +3 hours, +6 hours) and then use the time control to animate through 24 hours of the selected model, comparing the model visualisation with a display of the current tide cycle at that time, shown within the *Time Control* pop-up. The time control is also linked to the *Significant Assets* window which is populated with the list of man-made assets (road or road sections, buildings, etc.) which are potentially endangered by a flooding event at the period of time defined by time control slider. By selecting different models and forecast periods, decision makers can ultimately compare various flood development scenarios and thus improve risk management procedures.

Development of the flood application is ongoing and forthcoming improvements include a tool for selecting two sources of data, discovered through the semantic registry, and creating a dynamic request to the semantic integration service to combine them, thus giving end users the ability to produce new integrated data sources. The tool is also being demonstrated to, and will be evaluated by, potential users for the Solent region.

## 6. Conclusions

We have presented a semantic sensor web architecture that comprises a core collection of services that form a service-oriented architecture for data publication, discovery, and integration. The architecture also defines a resource-oriented user-facing web service exposing sensor readings as a linked data resource. The semantic sensor web architecture supports the four capabilities identified in Section 1: (1) identify relevant sources of data; (2) access sensor data in near real-time; (3) correlate disparate heterogeneous sources; and (4) support multiple conceptualisations.

The presented semantic sensor web can be used in conjunction with the services of the Open Geospatial Consortium Sensor Web Enablement (ogc-swe) [9], as described in Section 4 and depicted in Figure 3: data published through ogc-swe services can be incorporated into our semantic sensor web and data resulting from the semantic sensor web can feed into higher-level ogc-swe services. However, the semantic sensor web architecture presented goes beyond ogc-swe in several important ways. First, it supports data discovery based on semantic descriptions of the services and the datasets that they publish. This enables datasets to be identified based on their content, *i.e.*, their spatiotemporal and thematic coverage, rather than simply matching syntactic strings. Additionally, the relationships between concepts in the ontologies can be exploited for discovering suitable services and datasets. Second, it assists in the integration of heterogeneous datasets from autonomous providers by giving rise to semantically coherent models over the data. This enables the creation of *virtual* datasets that combine data from multiple sources, and the discovery of relationships in the virtual dataset. Note that each set of data may have been published according to a different conceptualisation. The integrator enables these to be related into a coherent conceptualisation. Third, it enables arbitrary models over the data to be used, *i.e.*, the service interfaces are not tied to the model used. The service interfaces are generic, *i.e.*, the operations supported, and their parameters, are not defined in terms of the underlying data model or representation. Instead, declarative query languages are used to express the data requirements. We note that version 2 of ogc-swe is moving in this direction with the definition of the eventing service [31]. This ensures that the semantic sensor web is generic and extensible, and can be used in applications beyond environmental monitoring, e.g., supply chain monitoring or healthcare, without redefining and implementing the services. Finally, the semantic sensor web architecture supports push-based delivery of data in a timely fashion, directly from the data source. Again, we note that ogc-swe version 2 is following a similar approach of using the Web Services Base Notification standard [8], although it requires a separate service from the data publication service (viz. sos) [31].

An example web application has been presented which uses the presented semantic sensor web to deliver a semantically-enabled and dynamically-constructed environmental decision support and flood emergency management tool. The tool is self-configuring in so far as it is based on the role and area of interest specified by the user. The tool enables a role-specific dynamic decision support environment to be experienced by the user. Semantic registry queries are used to discover relevant sources of real-time and historic sensor observations, modelled variables including flood forecasts, and other contextual data. New information sources can be created through the integration service by combining historic and real time sensor measurements, model outputs and a variety of spatially-enabled socio-economic variables. In addition to the use of open standards, the exclusive use of open source technologies without recourse to proprietary software makes the methodology particularly suitable for use by decision makers with limited resources and budget constraints and in rapid response to new conditions, e.g., in an emergency response scenario. The application is not specifically tied to the south of England, or to flood response planning, and can be implemented for sensor networks deployed anywhere in the world.

## Figures and Tables

**Table 1.** Functionality provided by the service interfaces. The use of existing standard interfaces is depicted in brackets. (Note that a mapping can be passed into the integration interface which relates the models of the data provided by the data sources to the global model of the data used by the integrated data source.)

**Interface**	**Functionality**
Service Metadata	Retrieve a description of the service, its functionality, available dataset(s) and their descriptions
Registration	Store a description of a service, its functionality, available dataset(s) and their descriptions
Integration	Enables the creation of an integrated dataset from one or more existing datasets
Query (ws-dai)	Enables a declarative query, in a supported query language, to be posed against the available data
Data Access (ws-dai)	Supports iterating over a dataset, which may have been generated in response to a query
Subscription (ws-n)	Supports requests to be sent notifications of new data items
Notification (ws-n)	Supports the receiving of notification messages

**Table 2.** Details of the deployed semantic sensor web services: the service name, the type of service, the interfaces supported, and the endpoint reference. The full list of active service endpoint references is available from http://www.semsorgrid4env.eu/index.php/services-applications (URL accessed 24 June 2011).

**Name**	**Service Type**	**Interfaces**	**Endpoint Reference**
Strabon	Semantic Registry Service	Service Metadata, Registration, Query, Data Access	http://www.semsorgrid4env.eu/services/registry-service
cco-ws	Streaming Data Service	Service Metadata, Data Access, Subscription	http://www.semsorgrid4env.eu/services/cco-stream
abp-ws	Streaming Data Service	Service Metadata, Data Access, Subscription	http://www.semsorgrid4env.eu/services/abp
snee-ws	Streaming Data Service	Service Metadata, Integration, Query, Data Access, Subscription	http://www.semsorgrid4env.eu/services/snee-stream
cco-stored	Stored Data Service	Service Metadata, Query, Data Access	http://www.semsorgrid4env.eu/services/cco-stored
iqs	Semantic Integrator Service	Service Metadata, Integration, Query, Data Access, Subscription	http://www.semsorgrid4env.eu/services/semantic-integrator
hlapi	Application Service	rest interface supporting http get	http://id.semsorgrid.ecs.soton.ac.uk/sensors/all

**Table 3.** User groups for the flood web application.

**User Group**	**Role**
Coastal Zone Manager	Management of environmental quality, flood risk management strategy, preparation and response and infrastructure management.
Flood Modeller	Development of scenarios of hazards and risk. Generation of wave over-topping, flood envelopes and prediction of potential assets at risk.
Emergency Services	Response to assets/population at risk, early warning and alert services, defence rescue and evacuation.
Ports Authority	Management of port operations, pilotage services and risk management.
General Public	Interest in potential flooding events and how they will affect them.
